# Aortic valve assessment by cardiac tomography: application in clinical practice

**DOI:** 10.47487/apcyccv.v6i3.516

**Published:** 2025-09-24

**Authors:** Danilo Weir-Restrepo, Andrés Sanchez-Muñoz, David Aristizábal-Colorado, Santiago Sierra-Castillo, Andrés Nicolás Arteaga Arellano, María Isabel Carvajal-Vélez, Pedro Abad-Díaz

**Affiliations:** 1 Departamento Cardiología, Universidad CES. Medellín, Antioquia - Colombia. Universidad CES Departamento Cardiología Universidad CES Medellín Antioquia Colombia; 2 Facultad de Medicina. Fundación Universitaria San Martín. Sabaneta, Antioquia-Colombia. Facultad de Medicina Fundación Universitaria San Martín Sabaneta Antioquia Colombia; 3 Departamento de Cardiología. Universidad de Antioquia, Medellín Colombia. Universidad de Antioquia Departamento de Cardiología Universidad de Antioquia Medellín Colombia; 4 Departamento de investigación e innovación, Universidad CES, Medellín, Antioquia - Colombia. Universidad CES Departamento de investigación e innovación Universidad CES Medellín Antioquia Colombia; 5 Departamento de Medicina Interna, Universidad Internacional del Ecuador (UIDE), AXXIS Hospital de Especialidades, Quito, Ecuador. Universidad Internacional del Ecuador Departamento de Medicina Interna Universidad Internacional del Ecuador (UIDE) AXXIS Hospital de Especialidades Quito Ecuador; 6 Departamento de Radiología - Imágenes Cardiovasculares, Ayudas diagnósticas SURA. Medellín, Antioquia - Colombia. Departamento de Radiología - Imágenes Cardiovasculares Ayudas diagnósticas SURA Medellín Antioquia Colombia; 7 Departamento de Radiología, Universidad CES. Medellín, Antioquia - Colombia. Universidad CES Departamento de Radiología Universidad CES Medellín Antioquia Colombia

**Keywords:** Aortic Valve, Cardiac Tomography, Aortic Stenosis, Endocarditis, Aortic Valve Insufficiency, Válvula Aórtica, Tomografía Cardíaca, Estenosis de la Válvula Aórtica, Endocarditis, Insuficiencia de la Válvula Aórtica

## Abstract

Aortic valve disease is a highly prevalent and clinically significant condition in the general population. Cardiac computed tomography (CT) has emerged as a widely available imaging modality that provides high-resolution static and dynamic information, enabling comprehensive evaluation of valve anatomy and function. This technique complements echocardiography and other imaging tools, adding incremental value to clinical decision-making. Its principal applications include identifying the etiology of valvular disease, grading severity, and assessing adjacent structures, all of which are critical for therapeutic planning. This non-systematic review synthesizes the evidence on the role of CT in the assessment of the aortic valve, focusing on aortic stenosis, aortic regurgitation, infective endocarditis, and postoperative evaluation. The current body of evidence underscores the expanding role of CT in the integrated diagnosis and longitudinal management of aortic valve disease.

## RESUMEN

La enfermedad valvular aórtica es un problema prevalente en la población general. La tomografía computarizada de válvulas cardíacas es una herramienta disponible para su evaluación, que permite obtener imágenes estáticas y dinámicas, proporcionando una valoración detallada de las características anatómicas y funcionales. Esta técnica puede complementar otros métodos de imagen, contribuyendo así a una toma de decisiones más informada. Una de sus principales utilidades radica en su capacidad para diagnosticar la etiología de la valvulopatía, determinar su gravedad y evaluar el estado de las estructuras adyacentes. Se realizó una revisión no sistemática de la literatura en la que se exploran las características de la estructura anatómica y la técnica de imagen, abordando además la estenosis aórtica, la regurgitación aórtica, la endocarditis infecciosa y la evaluación postquirúrgica de la válvula aórtica mediante este método de imagen. Consideramos importante destacar el potencial de esta técnica en el estudio de las afecciones valvulares aórticas.

## Introduction

Valvular heart disease, particularly involving the aortic valve, is a common clinical problem with multiple etiologies. The estimated prevalence of aortic stenosis in patients older than 75 years ranges from 2.6% to 22.8%; when adjusted, it is estimated at 12.4%, with severe disease present in 3.4% of cases, and up to 75.6% of these patients are symptomatic. ^1^ This condition encompasses both congenital and acquired causes and represents a major indication for surgical interventions across countries with different income levels. In fact, based on data from the United States, around 25,000 transcatheter aortic valve replacements (TAVR) were performed in 2015; by 2020, the figure had risen to approximately 100,000, and although no official data are yet available, it is estimated that by 2025, more than 280,000 such procedures will be performed. ^2^^,^^3^

Cardiac valve computed tomography (CT) is gaining importance due to its availability and its ability to provide detailed anatomical and pathological information, particularly in patients with inconclusive transthoracic echocardiography results. This technique has proven to be highly useful for clinical decision-making, especially before TAVR procedures. ^4^ In this review, we discuss the CT features of the aortic valve, highlight its main clinical applications, and outline additional considerations to support clinical decision-making.

## Methods

A non-systematic search was conducted up to September 2024 in PubMed, Scopus, and SciELO using the following terms: “aortic valve AND tomography”, “aortic stenosis AND tomography”, “aortic valve regurgitation AND tomography”, “aortic valve AND endocarditis AND tomography”, and “aortic valve AND tomography AND assessment”. The initial results yielded 8318, 12,138, and 36 records from the respective databases. These were subsequently filtered by the availability of full-text access. Duplicate records, articles published in languages other than Spanish or English, and letters to the editor were excluded. The remaining studies were assessed for relevance based on title and abstract and were finally included according to qualitative criteria defined by the authors, which formed the basis for the construction of this review.

### Anatomical structure

The aortic valve complex consists of the annulus, commissures, sinuses of Valsalva, coronary ostia, and the sinotubular junction. ^5^ This structure is composed of three cusps: the right and left cusps, which are directly connected to the coronary arteries, and a third cusp known as the non-coronary cusp. However, morphological variations may occur, with the presence of one to four aortic cusps. ^6^ The aortic valve is directly related to the left ventricular outflow tract, functioning as a unidirectional “hinge-like” valve. Together, these components form the functional structure of the aortic valve. ^5^

### Imaging technique and acquisition protocol

**Figure 1:**
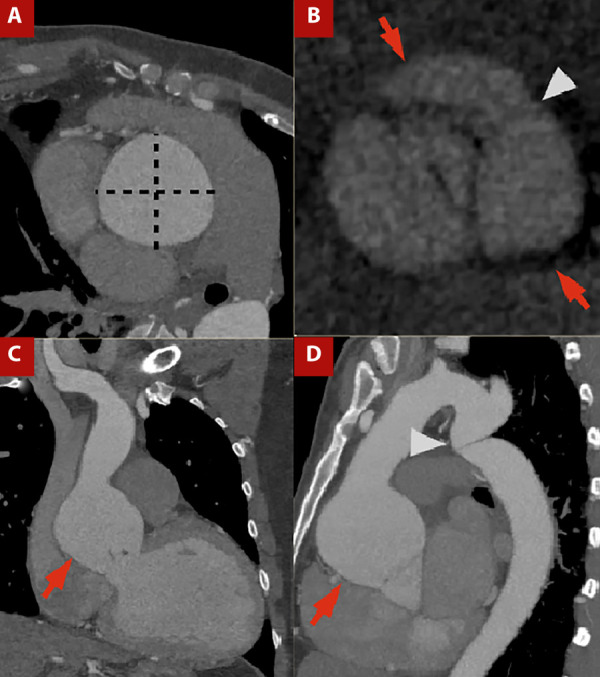
A) Contrast-enhanced CT angiography of the thoracic aorta with cardiac gating, orthogonal plane of the ascending aorta. B) Aortic valve plane in ventricular systole. C) Coronal view and D) Sagittal view.Ascending aortic dilatation is evident, with a maximum diameter of 64 × 64 mm measured in the orthogonal plane (dashed line in A), involving the sinotubular junction (arrows in C and D). A bicuspid aortic valve is present, with fusion of the right and left coronary sinuses (arrows in C) and visualization of the raphe (arrowhead in C). The patient also had thoracicaortic coarctation (arrowhead in D).

For morphological assessment of the aortic valve using cardiac CT, image acquisition requires cardiac synchronization and a retrospective protocol to capture the entire cardiac cycle, with a field of view (FOV) centered on the heart and aortic root, enabling evaluation of the aortic valve and adjacent structures such as the ascending aorta (Figure 1).

From a technical perspective, scanners with at least 64 detector rows should be used, aiming for slice thicknesses of 0.5-0.75 mm to achieve high spatial resolution; however, this may vary depending on heart rate (HR) and patient body habitus. ^7^^,^^8^^)^ In many protocols, beta-blockers are recommended to achieve a target HR <75 bpm, reducing cardiac motion artifacts over the aortic root and ensuring diagnostic image quality. ^4^ It should be noted, however, that in certain patients, such as those with symptomatic severe aortic stenosis or complications including infective endocarditis, the use of beta-blockers is contraindicated due to their effects on myocardial contractility and cardiac conduction. In such cases, alternative HR control strategies have been developed to optimize image acquisition. Among them, ivabradine has emerged as an effective option, as it lowers HR without affecting myocardial contractility, atrioventricular conduction, or blood pressure. ^7^ Indeed, in a retrospective study including nearly 6000 patients comparing ivabradine versus metoprolol for achieving target HR, ivabradine-based protocols were successful in a higher proportion of cases than metoprolol alone (89% vs. 77%; p<0.01), albeit with a longer interval from drug administration to CT acquisition (77 vs. 48 min; p<0.01). ^9^

Intravenous administration of high-concentration contrast medium (350-370 mg/cc) is required for optimal opacification, with doses ranging from 0.8 to 2.0 mL/kg depending on the clinical context. ^7^

For a detailed assessment of the valvular apparatus, reconstruction of ten cardiac phases spaced throughout the cardiac cycle is performed. This acquisition protocol facilitates accurate evaluation of valve anatomy and function, valve opening area, masses, prosthetic valves, and postoperative periprosthetic complications. It is important to note that ECG-gated acquisition protocols involve higher radiation exposure compared with conventional CT. Nevertheless, the advent of newer technologies has enabled acquisitions with lower radiation doses while maintaining high technical and diagnostic quality. ^10^

### Aortic valve assessment and planimetry

Cardiac CT enables precise measurement of the cusp opening area (mm²), the aortic valve area in mid-to-late systole, and the left ventricular outflow tract (LVOT). Notably, CT-derived and echocardiographic measurements are not interchangeable, reflecting the intrinsic nature of each modality. CT quantifies the anatomical valve area, often yielding larger values due to the inclusion of calcified structures and the absence of hemodynamic dependence, whereas echocardiography estimates the effective orifice area using the continuity equation, thus reflecting functional severity more closely. Accordingly, both techniques should be regarded as complementary. ^11^^,^^12^

Accurate measurement relies on planimetry, which requires alignment of three orthogonal axes through the LVOT to obtain an en face view of the aortic valve and allow assessment of cusp morphology, valve area, and eccentricity index. This is achieved by first identifying the cusp insertion plane in the coronal view and then aligning the orthogonal planes at the same level, generating a cross-sectional view of the valve apparatus (Figure 1A, 1B). ^11^

In clinical practice, CT assessment of the aortic valve is particularly valuable in the evaluation of aortic stenosis, defined as obstruction of LVOT flow at or near the valve level. Progressive cusp thickening initially causes trivial obstruction but may advance to require hemodynamic compensation, including aortic dilation and concentric left ventricular hypertrophy. ^5^^,^^13^

### Valvular calcium score

Calcium deposits in the aortic valve lead to a reduction in valve area and stiffness in its opening, with the extent of valvular calcification directly proportional to the severity of stenosis. CT has the advantage of not only detecting calcification but also quantifying it, which is particularly useful in patients with severe low-flow, low-gradient, or discordant stenosis, as the calcium score is a strong predictor of major adverse cardiovascular events (MACE). ^14^^,^^15^

**Figure 2:**
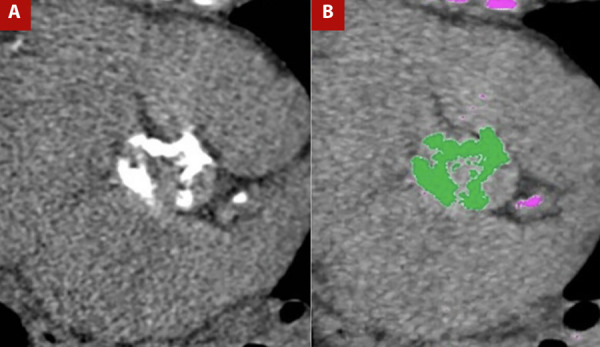
A) Non-contrast cardiac-gated CT in the aortic valve plane for calcium assessment and B) Post-processing for calcium quantification. Marked calcifications are present in the aortic valve leaflets, yielding a calcium score of 2889 Agatston units, highly suggestive of severe aortic valve stenosis.

Valvular calcium is assessed by non-contrast CT, typically performed at 120-140 kV (although other manufacturers provide different specifications), with a matrix size of 512 × 512, tube current adjusted according to body weight, slice thickness of 2.5-3 mm, images acquired during diastole, and calcifications defined as areas with density ≥130 Hounsfield units (HU). This protocol is known as the Agatston method. Based on HU values, findings are stratified into four groups: group 1, 130-199 HU; group 2, 200-299 HU; group 3, 300-399 HU; and group 4, ≥400 HU. The group number is multiplied by the area (cm²) of the lesion, and the values for all lesions are summed to obtain the calcium score, expressed as Agatston units (AU; Figure 2). Lesions in valve cusps and the annulus should be included, whereas calcifications of the left ventricular outflow tract, mitral valve, and proximal coronary arteries must be excluded to avoid overestimation. ^15^^,^^16^

The clinical utility of valvular calcium measurement has been extensively studied. Even after adjusting for cardiovascular and coronary risk factors, the degree of calcification is an independent predictor of cardiovascular and all-cause mortality. ^16^ It has been established as a predictor of mortality and the need for aortic valve replacement, with thresholds of 1377 AU for women and 2062 AU for men. The area under the curve for these thresholds is 0.92 in women and 0.89 in men, with sensitivities of 88% and 81% and specificities of 81% and 87%, respectively, for severe aortic stenosis ^17^. To optimize the definition of severe aortic stenosis, cut-off levels have been proposed and are presented in Table 1. ^16^

**Table 1:** Cut-off values of aortic valve calcium score for the probability of severe aortic stenosis.

Probability of severe aortic stenosis	Men	Women
Unlikely	<1600 AU	<800 AU
Probable	> 2000 AU	>1200 AU
Highly probable	> 3000 AU	> 1600 AU

AU: Agatston Units.

In addition, this technique can aid in procedural planning, providing valuable information for determining the surgical approach, whether valve replacement alone or combined with ascending aortic repair, among other technical considerations, and in assessing suitability for transcatheter aortic valve implantation (TAVI). ^10^^,^^14^^,^^15^^,^^18^ Specifically, in the evaluation of aortic calcification, certain features favor TAVI over open surgery, including extensive calcification of the ascending aorta (porcelain aorta), anatomical features that make surgical access challenging (severe calcification or tortuosity), and the absence of other concomitant procedures. A major advantage of TAVI is its feasibility in comorbid patients at high surgical risk. ^17^^,^^19^

### Pre-TAVI protocol

**Figure 3:**
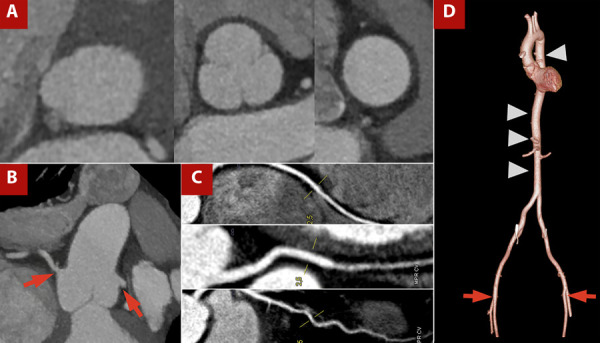
TAVI protocol CT angiography with a one-step approach, including coronary CT angiography. **A)** Orthogonal measurements at the aortic valve plane, sinuses of Valsalva, and sinotubular junction. **B)** Assessment of coronary ostial height and origin (red arrow). **C)** Curved multiplanar reconstruction of coronary arteries showing atherosclerotic disease. **D)** 3D reconstruction of the thoracoabdominal aorta (arrowhead) and femoral access routes (red arrow).

In relation to TAVI, coronary artery disease must be excluded as part of the pre-procedural protocol, and patients are usually referred for coronary angiography. However, some protocols allow coronary CT angiography to rule out significant coronary disease with adequate sensitivity and specificity. In these centers, valve and coronary assessment are performed with CT in TAVI candidates, and the need for invasive studies such as angiography is determined on the basis of these findings, an approach known as the one-step strategy. ^20^ Figure 3 shows an example of images acquired using the pre-TAVI protocol.

Several parameters are evaluated, including annular ellipticity, the tubular index of the left ventricular outflow tract, coronary height, virtual transcatheter valve-to-coronary (VTC) distance, membranous septum length, and grading of calcification, together with details on the aortic root, valve dimensions, and annular measurements. ^19^^,^^20^ CT also enables assessment of the peripheral vasculature, providing information on the aorta and iliofemoral vessels, as well as the burden of atherosclerosis. These data support interventional teams in planning the access route, guiding post-implantation follow-up, and predicting the risk of complications such as annular rupture, conduction disturbances, coronary occlusion, aortic injury, and vascular complications, issues that go beyond the scope of this review. ^19^^,^^20^

## Other applications

### Aortic regurgitation

Aortic insufficiency, or aortic valve regurgitation, is characterized by retrograde blood flow from the aorta into the left ventricle due to inadequate adaptation of the valve during diastole, caused by incomplete cusp closure. Various conditions can lead to this disorder through progressive valvular stiffening or distortion of the aortic root. ^21^

On CT, malcoaptation of the cusps can be visualized in late-diastolic phases. However, owing to the lower temporal resolution of CT compared with echocardiography, echocardiography remains the diagnostic tool of choice for evaluating aortic regurgitation. Additional findings depend on the underlying etiology, including shortened and thickened cusps, aortic root dilation, and left ventricular hypertrophy, among others. It is worth noting that CT assessment is limited, as regurgitant volumes cannot be accurately quantified, restricting its role to purely anatomical evaluation. ^4^^,^^22^

### Infective endocarditis

Although echocardiography is the most widely used imaging technique for evaluating this condition, CT can play a valuable role when echocardiographic findings are inconclusive, as it provides additional anatomical information such as the location of the infectious focus, abscesses, and pseudoaneurysms. Furthermore, when combined with positron emission tomography, CT can offer added diagnostic and prognostic value for cardiovascular events. ^23^ Indeed, the latest European Society of Cardiology guidelines on infective endocarditis establish CT as the principal imaging modality for diagnosing perivalvular complications. ^24^

CT findings in infective endocarditis are mainly related to perivalvular complications, as the technique allows assessment of the perivalvular extent of infection. Such complications include pseudoaneurysm, dehiscence, fistulae, and abscesses; the latter typically presents as fluid collections with densities of 20-50 HU or as heterogeneous collections around the valve. A hyperintense ring in late phases can also be observed. ^25^^,^^26^

### Post-surgical assessment

For the evaluation of prosthetic valves after surgery, CT allows high-resolution imaging with minimal motion artifacts, enabling adequate visualization of valve structure and function as well as the perivalvular space, and thus the identification of potential surgical complications. However, the technique has lower performance in the assessment of bioprosthetic valves. ^27^

The main post-surgical complications following the implantation of these valves are prosthetic valve obstruction, paravalvular leak, thrombus, prosthesis-patient mismatch, endocarditis, and pseudoaneurysm, which represent the principal indications for requesting CT imaging. ^28^

Prosthetic valve obstruction is typically observed as restricted opening angles (e.g., 20°) in the most commonly used valves (such as the SJM Regent mechanical valve) in the aortic position, together with a reduced aortic valve area. Pannus usually appears as a linear, low-attenuation filling defect located just beneath the valve ring and adjacent to the left ventricular wall, and it can usually be distinguished from thrombus, which presents as a low-attenuation filling defect on the aortic side of an aortic prosthetic valve. Obstructive thrombi appear as irregular hypodense masses directly adherent to the cusps and hinge points, causing mechanical obstruction by limiting cusp motion. ^28^

Paravalvular leak is identified as a separation between the native valve annulus and the prosthetic valve. In patients with suspected prosthetic valve regurgitation, CT can be useful for excluding abscess formation and for providing information relevant to treatment planning. ^28^

**Figure 4:**
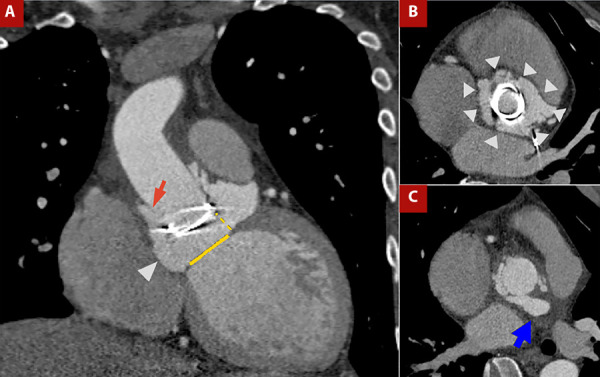
A) Contrast-enhanced CT angiography of the thoracic aorta with cardiac gating, coronal view. B) Orthogonal plane at the aortic root and C) Ascending aorta. Patient with Marfan syndrome, prior aortic valve prosthesis, and Bentall procedure, now presenting with prosthetic valve endocarditis. Findings include migration of the prosthetic valve (yellow dashed line in A) from the aortic valve plane (yellow solid line in A), dehiscence of the vascular graft (red arrow in A), circumferential (360°) paravalvular leak surrounding the prosthesis (arrowheads in A and B), and a retroaortic paravalvular abscess (blue arrow in C).

Figure 4 shows the case of a patient with prosthetic valve infective endocarditis, in whom CT demonstrated prosthetic valve migration, vascular graft dehiscence with paravalvular leak, and a retroaortic paravalvular abscess.

**Table 2:** Main indications for cardiac computed tomography in aortic valve disease

Indications
Suspected aortic stenosis
Inconclusive echocardiography or technical limitations (e.g., obese patients)
Pre-procedural assessment before transcatheter aortic valve replacement
One-step strategy with cardiac CT and coronary CT angiography before intervention
Suspected aortic valve regurgitation associated with another pathological process
Suspected complications related to infective endocarditis
Suspected complications following aortic valve procedures

## Conclusions

Cardiac CT is a valuable tool for the evaluation of aortic pathologies. It is now widely available and can be particularly useful in cases where transthoracic echocardiography yields inconclusive results. The main indications for cardiac CT in aortic disease are summarized in Table 2. This imaging modality provides a detailed morphological assessment of the aortic valve and adjacent structures, thereby supporting clinical decision-making, particularly before TAVR. Furthermore, the quantification of valvular calcium by CT can serve as a predictor of mortality and the need for aortic valve replacement. Its applicability, therefore, extends across a range of clinical scenarios beyond primary valvular disease, offering adequate diagnostic performance to guide therapeutic strategies. Nonetheless, it should be emphasized that high-quality imaging requires appropriate technical resources and specific protocols to minimize artifacts.
